# Table Talk: revision of an observational tool to characterize the feeding environment in early care and education settings

**DOI:** 10.1186/s12889-020-10087-8

**Published:** 2021-01-07

**Authors:** Taren Swindle, Josh Phelps, Nicole M. McBride, James P. Selig, Julie M. Rutledge, Swapna Manyam

**Affiliations:** 1 Department of Family and Preventive Medicine, University of Arkansas for Medical Sciences, 4301 W. Markham St, #530, Little Rock, AR 72205-7199 USA; 2 Department of Dietetics and Nutrition, University of Arkansas for Medical Sciences, 4301 W. Markham St, #530, Little Rock, AR 72205-7199 USA; 3 College of Public Health, Department of Biostatistics, University of Arkansas for Medical Sciences, 4301 W. Markham St, #530, Little Rock, AR 72205-7199 USA; 4 School of Human Ecology, Louisiana Tech University, P.O. Box 3167, Ruston, LA 71272 USA

**Keywords:** Early childhood educators, Childcare teachers, Feeding, Childcare, Meals, Communication

## Abstract

**Objective:**

The Table Talk tool is an observational assessment of early care and education teacher (ECET) mealtime practices. The Table Talk Revised (TT-R) tool incorporates new constructs that emerged from qualitative research and teases apart existing categories to improve nuance of data capture. The objective of this study was to evaluate the TT-R, document interrater reliability for the TT-R, and report on ECET feeding communications in broader settings than previously studied (i.e., beyond a single Lunch and Head Start only).

**Methods:**

Trained observers conducted mealtime observations in classrooms (*N*_*classroms*_ = 63, 10 sites) during Breakfast and two Lunches for both Lead and Assistant ECETs (*N* = 126). Classrooms were spread across Head Start in an urban area (60%), Head Starts in a rural area (24%), and a state-funded preschool (16%).

**Results:**

On average, there were 22.17 (SD = 10.92) total verbal feeding communications at Breakfast, 37.72 (SD = 15.83) at Lunch_1_, and 34.39 (SD = 15.05) at Lunch_2_ with meals averaging 25 min. The most commonly observed supportive statement category was Exploring Foods for Lead (Breakfast = 1.61, Lunch_1_ = 3.23, Lunch_2_ = 2.70) and Assistant ECETs (Breakfast = .89, Lunch_1_ = 2.03) except for Lunch_2_ which was Encourages Trying in a Positive Way (Lunch_2_ = 1.30). The most commonly observed unsupportive statement category was Firm Behavioral Control for both Lead (Breakfast = 3.61, Lunch_1_ = 5.84, Lunch_2_ = 5.51) and Assistants ECETs (Breakfast = 3.11, Lunch_1_ = 6.38, Lunch_2_ = 4.32). The majority of Interclass Correlation Coefficients indicating interrater reliability were in the excellent range (64%) for commonly occurring statement categories, and 14 of the 19 low frequency statement categories had > 80% agreement.

**Conclusions and implications:**

Overall, items added to the Table Talk tool performed well, and interrater reliability was favorable. Our study also documented differences between Lead and Assistant teachers in mealtime practices and illustrated differing patterns of interaction between lunches and breakfast, important findings to inform future research and practice. The TT-R may be a useful measurement tool for monitoring and evaluating ECET practices in mealtime environments as well as informing intervention.

**Supplementary Information:**

The online version contains supplementary material available at 10.1186/s12889-020-10087-8.

## Introduction

Children’s dietary habits before the age of 5 years may influence nutrition behaviors and weight outcomes later in life as well as long-term health [1, 2]. Nutrition behaviors (e.g., dietary habits) and weight outcomes (e.g., obesity and overweight) among children are, in part, a result of their interaction with social factors [3]. Specifically, genetic predispositions may interact with a number of co-existing environmental factors to impact weight outcomes [3]. The bioecological theory recognizes that “human development results from the interplay of *Process x Person x Context x Time.*” [4] One potentially important environmental factor toward influencing nutrition behaviors and weight outcomes among children is exposure to early care and education (ECE) settings (i.e., childcare) [5, 6].

Early care and education settings serve more than 50% of children under the age of 5 across the United States; the majority of children in ECE spend 35 h or more per week at these centers [7]. Within these environments, whether in federally -funded programs [i.e., Head Start (HS)], parish/state-funded programs, or private programs, there are standards of practice/care associated with children’s development, health, and well-being (e.g., U.S. Department of Health & Human Services. Head Start - Early Childhood Learning & Knowledge Center). As healthy children are better learners, these standards focus on engaging preschool children in activities to learn about healthy and unhealthy foods, with the understanding that some foods are healthier than others (e.g., Louisiana’s Birth to Five - Early Learning & Development Standards, 2013).

Standards and recommendations also exist for feeding practices by adults in ECE settings. This is because the ECE eating environment may contribute to children developing healthy feeding behaviors. In particular, the behaviors and practices of early care and education teachers (ECETs) may influence which feeding behaviors children learn in the short-term (for example, within the classroom), and the feeding behaviors children engage in over the long-term (for example, behaviors carried with them beyond the classroom environment as they get older) [5]. ECETs have an important role of modeling responsive and supportive feeding practices and behaviors toward creating a positive mealtime environment, which includes allowing “children to choose which and how much … foods offered they will eat,” encouraging children “to listen to their hunger and fullness cues,” and avoiding coercive feeding practices, such as the “use of food as a reward or punishment” [8]. Coercive practices (e.g., pressuring children to eat) function to “teach children to eat for reasons unrelated to appetite and, hence, more than they need and fail to support development of healthy food preferences and appetite regulation,” potentially increasing risk for development of chronic diseases later in life [9]. Thus, ECETs play an important role in creating formative mealtime environments for children.

Measuring feeding practices at ECE mealtimes is crucial to inform interventions that improve children’s diets in both the short and long term. In order to identify, measure, and assess change in important variables (supportive and/or coercive feeding practices and behaviors), appropriate assessment tools are necessary. A key consideration is the use of self-report versus observational measures. Fallon et al. (2018) compared findings of observed feeding practices (via the Environment and Policy Assessment and Observation –Expanded Feeding Practices tool, EPAO-EFP) to self-reported feeding practices (adapted questionnaire from the EPAO-Self Report) of HS teachers [10]. These authors found there was “a higher level of agreement” between self-reported and observed practices regarding controlling (“highly discouraged”) feeding practices (“78.8–97.6% agreement”) than healthful feeding practices (“11.8–20.0% agreement”) of HS teachers. Based on these findings, it appears HS teachers may be overestimating their abilities to use healthful feeding practices when engaging with children during meal times, while more accurately reporting detrimental feeding practices in their self-report. An additional study comparing teacher-reported fidelity practices (e.g., role modeling) in a nutrition intervention to observed practices reported low concordance [11]. Together, these findings highlight the need for measurement tools appropriate for real-time examination of teacher feeding practices beyond self-report.

Having an accurate observational assessment of ECET feeding practices is essential to developing and delivering effective training to promote healthful feeding practices in the ECE setting. An often studied, modified, and reported tool is the Environmental Policy Assessment and Observation (EPAO) survey [12]. Most recently, Fallon et al. (2018) described a tool based on an expanded version of the nutrition practice-related items from the EPAO resulting in a tool used for real-time examination of “staff feeding practices,” the EPAO-EFP. Regarding internal consistency of mealtime behavior items in the EPAO-EFP, a Cronbach’s alpha of 0.70 revealed “adequate reliability.” [10, 13] Inter-rater reliability was assessed as Kappa = 0.83 and Kappa = 0.84 at the beginning and later in the study, respectively [10, 13–15].

Another option for a real-time observational tool of ECET feeding practices is Table Talk, a tool designed for use by trained health professionals, researchers, and early childhood professionals. This tool is intended to provide detailed information on ECETs’ ‘table talk’ that can be used to inform training and resources for ECETs to improve use of supportive behaviours and communications while also decreasing use of unsupportive ones. A previous study of Table Talk focused on HS classrooms in Southern US states to capture aspects of ECET communication that contribute to a Positive Mealtime Environment (PME) *and* a Negative Mealtime Environment (NME) [16]. This study addressed development of the tool, properties of observed items, and test-retest reliability on a sub-sample. Real-time assessment of ECET feeding practices using the Table Talk tool allows for capture of feeding communications without the limitations of potential ceiling effects. Different from the EPAO-EFP, the Table Talk tool counts communications continuously and does not truncate observations with Likert scaling or yes/no responses. Further, communications of both Lead and Assistant ECETs are captured in real time. A full comparison of the EPAO-EFP and the Table Talk tool is found in Supplementary File 1.

In prior development of Table Talk, observations were limited to HS classrooms and data were reported for a single Lunch, but not Breakfast. Further, data on interrater reliability have not yet been reported. The Table Talk tool has since been revised and expanded to reflect (a) the authors’ experience using the tool and desire to refine a key category (positive comments) and (b) qualitative work by the authors that suggested other salient, intentional practices teachers use that were not being captured by the tool. We expected these revisions to provide a more nuanced and complete capture of mealtime in the ECE environment, including new concepts contributed by ECETs themselves. The objectives of the current paper are to: (a) evaluate a revised version of the Table Talk tool, (b) document interrater reliability for the revised Table Talk tool, and (c) report on ECET feeding communications at both Breakfast and Lunch in a broader setting than the prior study (i.e., beyond a single lunch in HS only).

## Methods

### Table talk

The original Table Talk tool was designed for immediate in-person coding of ECETs’ statements at mealtimes [16]. The tool reflects evidence-based practices and recommendations for mealtime interactions with children. The tool itself is partitioned into two parts; the tool’s top half contains the “positive, supportive” statement categories, and the bottom half of the tool contains the “negative, unsupportive” statement categories. From these general categories, the tool then directs for coding of the statements into discrete categories. The original tool had a total of 4 positive statement categories and 8 negative statement categories.

The Table Talk Revised (TT-R) tool used in this study was updated to include a total of 6 supportive statement categories and 9 unsupportive statement categories. Revisions were made before study observations, and updates were made to training protocols on these changes. The category “positive comments about the food served” from the original tool was broken into two different classifications in this wave: “Positive Comments- Teacher Focus” and “Positive Comments- Food Focus.” These two branches of positive comments were used to distinguish between comments that were attuned to the ECET’s positive opinion of the food (e.g., “These apples taste great to me!”) and comments that more focused on the positive characteristics of the food (e.g., “These apples are so bright and healthy!”). This reflects qualitative feedback that ECETS intentionally make food-focused comments when they do not prefer the food themselves [17]. Also new in the supportive section of the TT-R was the addition of the “Nutrition Coaching” statement category. This new category captures ECET statements about the meal that focused on an individual child’s experience. The addition of this category reflects qualitative work by the authors which uncovered how ECETs intentionally apply other classrooms skills (e.g., social-emotional support, emotion coaching) to supporting children at mealtimes (i.e., a whole day approach) [17]. Statements in this category help the children connect the current experience of the meal with other experiences they have had and guide children to use their senses to learn about the food (e.g., “Do you remember when we had carrots last week? Those were cooked, but these are raw.”). The primary change in the unsupportive section of the tool was the addition of the statement category “Social Comparison.” This change is supported by the presence of this construct in the Building Mealtime Environments and Relationships tool [18] as well as the presence of this strategy in recent qualitative work of the authors [17]. Statements in this category were those identified as comparing a child’s eating habits to those of another person’s or another person’s expectation of the child (e.g., “You should eat more like Sally.” “Your mom will be so happy you ate so much!”). Other than these three changes, the tool remained the same as its original design. All justifications and citations for original statement categories are included in the prior publication.

### Research design

The study team recruited centers consistent with published protocols for two implementation research studies [19, 20]. To assess the TT-R tool, trained observers (TOs) conducted mealtime observations between February 2018 and January 2019 from 126 ECETs in 63 classrooms across two states in the southern United States. In Arkansas (AR), there were 10 sites in an urban Head Start consisting of 38 classrooms across sites (City Population = 198,606); the other state (Louisiana, LA) had 2 sites – one Head Start with 15 classrooms and one state-funded preschool with 10 classrooms in a rural area (City Population = 22,234). All sites included in this study were full-day programs. Children in the classrooms included in the study were 3–5 years old. Demographic information was collected from the ECETs, and Table 1 displays demographics by ECET role (i.e., Lead or Assistant). The majority of ECETs were female (99%) and Black (73.3%); most identified as non-Hispanic (95.3%). Teachers were predominantly 41 years or older (61.4%), and about one third reported 11–20 years of teaching experience (35.6%) and had a college level education (31.1%). Observations were reflective of classrooms before the implementation of a nutrition education training and curriculum in AR; observations were after training in LA. Data collection was conducted in accordance with an approved IRB protocol, which was standardized across the two sites.

**Table 1:** Total sample teacher demographics and demographics by teacher type

	Total	Lead	Assistant
Female, % (*n*)	99% (102)	97.9% (47)	100% (55)
Age in years, % (*n*)			
19–24	4% (4)	2.2% (1)	5.5% (3)
25–34	20.8% (21)	15.2% (7)	25.5% (14)
35–40	13.9% (14)	19.6% (9)	9.1% (5)
41+	61.4% (62)	63% (29)	60% (33)
Race, % (*n*)			
Black	73.3% (77)	68.8% (33)	77.2% (44)
White	22.9% (24)	31.3% (15)	15.8% (9)
Asian	1.9% (2)	–	3.5% (2)
Other	1.9% (2)	–	3.5% (2)
Non-Hispanic, % (*n*)	95.3 (81)	97.1% (33)	94.1% (48)
Education level, % (*n*)			
HS/GED	10.4% (11)	–	19% (11)
Some college	19.8% (21)	–	36.2% (21)
Associates/CDA	31.1% (33)	22.9% (11)	37.9% (22)
Bachelor’s	30.2% (32)	62.5% (30)	3.4% (2)
Graduate degree	7.5% (8)	14.6% (7)	1.7% (1)
Teaching experience, % (*n*)			
< 1 year	2% (2)	–	3.6% (2)
1–10 years	31.7% (32)	32.6% (15)	30.9% (17)
11–20 years	35.6% (36)	34.8% (16)	36.4% (20)
21+ years	30.7% (31)	32.6% (15)	29.1% (16)

*Note*. Valid percentages displayed

### Observer training

The Primary Investigator (PI) trained observers on the data collection process via in-person meetings in both states. Within the initial meeting, observers were specifically trained on the intent of each TT-R positive and negative statement category, coding statements into discrete categories, and incorporating into a classroom setting with minimal disturbances. Training used pre-recorded videos of mealtimes. Observers in training compared their coding to a gold-standard observer (the PI, Co-PI, and 2 Research Associates). Gold-standard observers displayed above 90% consistency between each other in coding ECET statements in live settings. After the initial in-person training, observers viewed additional videos of mealtimes on their own time and coded ECET statements. The observers’ data were compared to those of a gold-standard observer. To be considered reliable, observers had to demonstrate only slight deviation from gold standard observers (± 1 for counts ≤3, ± 2 for counts > 3). At least 85% percent agreement on the videos was required before the observer was allowed to collect classroom data. All observers were accompanied on their first live classroom observation by a gold standard observer, where reliability was verified in the live setting. Observers (*N =* 22) included undergraduate psychology/human development and family science students, graduate dietetics students, and professionals from dietetics and public health.

### Data collection

In AR, data collection took place between February and May of 2018; LA data collection took place between October and January of 2019. Data were collected from two Lunch observations for all ECETs across both states, while a Breakfast observation was only collected from AR. Approximately 10 min before the scheduled mealtime, observers arrived and integrated into the classroom or cafeteria, striving to sit as far from the meal as possible while still able to hear the meal conversations. Observers focused on coding unique verbal interactions between ECETs and students into appropriate statement categories within the TT-R and noted the length of the meal from when the first child received food to the removal of food from the last child. Unique verbal interactions, not repeated statements within the same interaction (e.g., “Try your peaches. Try your peaches.”), were recorded as a single comment. Meals served in a classroom (*n* = 28) were watched by one observer coding statements from the Lead ECET and Assistant ECET. Up to 20 children were present at meals in the classroom. Meals served in a cafeteria setting (*n =* 12) were innately noisier and more difficult to code. Up to six classrooms were in a cafeteria at the same time for up to 120 children present at once. To help capture communications in this environment, classrooms in these settings were assigned two observers. In cafeteria settings, one observer coded statements from the Lead ECET, and the other coded statements from the Assistant ECET. The average time between Lunch observations for AR was 37 days and 82 for LA. All observation occasions had an approximate average duration of 25 min [Lunch_1_ *M* = 25.06, Lunch_2_ *M* = 25.06, Breakfast *M* = 23.87 (AR only)].

### Analyses

The researchers used descriptive statistics to summarize ECET demographics as well as individual TT-R statement categories and summary scores. For supportive and unsupportive statements, the researchers created summary counts by totaling the number of observed verbal ECET communications within these areas. Cases used to calculate interrater reliability (*n* = 11) represent comparison of field observations of a gold standard observer with a trained observer who had demonstrated video reliability but who had not yet demonstrated live reliability. Lunch mealtimes were used to assess reliability of the TT-R. Because the TT-R captures feeding communication counts continuously without truncating (i.e., such as with Likert scaling), inter-observer agreement (e.g., interrater reliability) was utilized rather than test-retest (of a single observer over time) to examine reliability.

Our approach to interrater reliability analysis was informed by best practices in psychometrics for direct observation [21, 22]. Percent agreement is appropriate to capture interrater reliability for low occurring (average count of the TT-R statement category < 1 across the observations) but salient items, although it does not account for chance agreement [23]. Statement category counts were dichotomized; statement categories occurring less than once on average were assigned 0; statement categories with 1 or more occurrences on average were assigned 1, and then the difference of the two ratings (gold standard observer and trained observer) was taken. Percent agreement is the number of absolute agreements of gold standard observer vs. trained observer rating divided by the total number of paired ratings [23]. Percent agreement values can be interpreted as the percent of data that are in agreement, e.g., 91% percent agreement would mean 10 out of 11 rating pairs had absolute agreement [23]. For more commonly occurring statement categories (average count of the TT-R statement category ≥1 across the three time points), interrater reliability was determined with intraclass correlation coefficients (ICC). ICC estimates were based on a mean rating (*k* = 2), absolute agreement, one way random-effects model [24]. Guidelines suggested by Koo and Li (2016) were used to interpret the ICC estimates as poor (< 0.5), moderate (0.5–0.75), good (0.75–0.90), and excellent (> 0.90). IBM Corp Statistical Package for the Social Sciences (SPSS) version 25.0 was used for all analyses.

## Results

### Evaluating TT-R

Table 2 summarizes average numbers of recorded feeding communications by Lead and Assistant ECETs across each time point. Added items were observed at both lunches and meals. The newly separated categories of Teacher Focus and Food Focus Positive Comments were observed about 2 and 1.5 times per lunch and about 1 and 0.5 times per breakfast, respectively. Assistant ECETs were more likely to use Teacher Focus comments than Food Focus comments. Lead ECETs used these two comment categories at similar rates. For the new category of Nutrition Coaching, Lead teachers used Nutrition Coaching about twice as often as Assistants did. Further, one class observation included 11 instances of Nutrition Coaching. For the additional unsupportive item of Social Comparisons, Lead and Assistant use were similar with about 0.60 uses at lunches and 0.5 at breakfast.

**Table 2:** Average number of communications by teacher type across three mealtime observations

	Lead Lunch 1	Lead Lunch 2	Lead Breakfast^a^	Assistant Lunch 1	Assistant Lunch 2	Assistant Breakfast^a^	Total Lunch 1	Total Lunch 2	Total Breakfast^a^
*Supportive Communications*	*M* (SD) Range								
Positive comments – Teacher Focus. *(*e.g.*, I am really enjoying the peas today. Yummy.)*	.90 (1.36) 0–5	1.33 (1.52) 0–6	.58 (0.95) 0–5	1.07 (1.20) 0–5	.88 (1.07) 0–4	.49 (0.78) 0–3	1.98 (2.17) 0–9	2.12 (1.93) 0–7	1.09 (1.34) 0–6
Positive comments – Food Focus *(*e.g.*, The food is so bright and colorful. We have all the food groups today!)*	.95 (1.42) 0–5	.98 (1.49) 0–7	.26 (0.60) 0–2	.57 (1.68) 0–11	.42 (0.82) 0–3	.17 (0.45)0–2	1.54 (2.51) 0–14	1.41 (1.83) 0–7	.46 (0.92) 0–3
Hunger cues *(Are you full? How does your belly feel?)*	.16 (.45) 0–2	.39 (1.19) 0–8	.13 (0.34) 0–1	.15 (0.48) 0–3	.16 (0.46) 0–2	.11 (0.53)0–3	.31 (0.81) 0–5	.56 (1.38) 0–8	.26 (0.61) 0–3
Encourage trying in positive way *(Would you like to try the peas?)*	1.73 (2.59) 0–14	1.70 (1.72) 0–7	.92 (1.38) 0–5	1.31 (1.87) 0–8	1.30 (1.56) 0–7	.80 (1.21) 0–5	3.05 (3.65) 0–21	2.95 (2.29) 0–8	1.80 (2.19) 0–9
Nutrition Coaching (focus on child experiences; *What does it smell like?Have you had this before?)*	1.24 (1.45) 0–6	1.44 (1.75) 0–8	1.16 (1.98) 0–7	.87 (1.36) 0–7	.70 (1.32) 0–8	.29 (0.62) 0–3	2.10 (2.34) 0–11	2.12 (2.04) 0–9	1.34 (2.01) 0–7
Exploring foods (focus on food itself; *What food group is this? Where does it grow? What are the ingredients?)*	3.23 (3.37) 0–18	2.70 (3.13) 0–13	1.61 (1.90) 0–6	2.03 (2.37) 0–9	1.14 (1.86) 0–10	.89 (1.39) 0–6	5.23 (4.37) 0–19	3.85 (3.92) 0–19	2.57 (2.69) 0–9
*Total Supportive Communications*	8.21 (7.53) 0–39	8.51 (6.03) 0–26	4.66 (4.58) 0–18	6.00 (5.50) 0–27	4.60 (4.15) 0–17	2.74 (2.91) 0–11	14.21 (10.79) 0–61	13.00 (7.31) 0–28	7.51 (6.56) 0–29
*Unsupportive Communications*	*M* (SD) Range								
Negative comments about the food served *(I can’t believe we’re having this again. I don’t like peas.)*	.05 (0.28) 0–2	.00 (0.00)0	.05 (0.23) 0–1	.08 (0.33) 0–2	.02 (0.13) 0–1	.03 (0.17) 0–1	.13 (0.50) 0–3	.02 (0.13) 0–1	.09 (0.28) 0–1
Pressure to eat. (*Eat your food. Take a bite. Clean your plate. Finish.)*	3.77 (3.72) 0–17	3.98 (3.39) 0–14	3.08 (2.76) 0–9	3.28 (2.92) 0–12	3.47 (3.75) 0–20	2.74 (2.56) 0–10	7.08 (5.06) 0–23	7.31 (5.55) 0–32	5.86 (4.39) 0–18
Hurries to finish eating *(We’re waiting on you. Let’s hurry so we can go to recess.)*	.95 (1.38) 0–6	1.15 (1.53) 0–7	.58 (0.89) 0–4	.52 (0.79) 0–3	.70 (1.20) 0–5	.49 (1.01) 0–4	1.49 (1.77) 0–7	1.81 (2.22) 0–10	1.03 (1.40) 0–4
Discourage manipulating food (*Eat; don’t play. That’s sticky and nasty.)*	.89 (1.45) 0–6	.98 (1.36) 0–7	.47 (0.86) 0–3	.69 (0.92) 0–3	.88 (1.23) 0–5	.26 (0.56) 0–2	1.59 (1.84) 0–7	1.80 (1.93) 0–8	.74 (1.25) 0–5
Social Comparison *(*e.g.*, Look how Susie is eating. Eat like her. Your mom is going to be sad.)*	.55 (1.52) 0–9	.25 (0.54) 0–2	.32 (0.62) 0–2	.11 (0.41) 0–2	.23 (0.60) 0–3	.17 (0.71) 0–4	.64 (1.65) 0–10	.46 (0.90) 0–4	.51 (1.17) 0–6
Threats *(to encourage eating)* *(If you don’t eat, you’ll be over here by yourself.)*	.02 (0.13) 0–1	.02 (0.13) 0–1	.00 (0.00) 0	.02 (0.13) 0–1	.04 (0.27) 0–2	.00 (0.00) 0	.03 (0.18) 0–1	.05 (0.29) 0–2	.00 (0.00) 0
Indicate preference for unhealthy food. *(I wish we were having french fries today.)*	.10 (0.35) 0–2	.02 (0.13) 0–1	.08 (0.36) 0–2	.16 (0.55) 0–3	.05 (0.29) 0–2	.06 (0.24) 0–1	.26 (0.84) 0–5	.10 (0.48) 0–3	.14 (0.55) 0–3
Food as a reward. *(If you eat your vegetable, you can have dessert).*	.08 (0.52) 0–4	.15 (0.44) 0–2	.03 (0.16) 0–1	.03 (0.18) 0–1	.02 (0.13) 0–1	.00 (0.00) 0	.05 (0.22) 0–1	.15 (0.49) 0–2	.00 (0.00) 0
Firm behavioral control *(Turn around. Sit up straight. Hands in your lap)*	5.84 (4.62) 0–20	5.51 (4.36) 0–17	3.61 (3.36) 0–14	6.38 (5.68) 0–26	4.32 (4.17) 0–23	3.11 (3.03) 0–15	12.23 (8.07) 0–46	9.73 (6.93) 0–39	6.72 (5.04) 0–24
*Total Unsupportive Communications*^*b*^	6.40 (5.72) 0–25	6.44 (4.83) 0–21	4.61 (3.82) 0–13	4.90 (3.57) 0–15	5.40 (4.67) 0–22	3.74 (2.93) 0–10	11.28 (7.31) 0–31	11.69 (6.91) 1–34	8.37 (5.83) 0–23
*Total Communications*^*c*^	20.45 (11.37) 0–52	20.13 (11.44) 0–55	12.87 (7.48) 0–31	17.28 (9.89) 0–45	14.32 (9.24) 0–41	9.42 (5.60) 0–23	37.72 (15.83) 10–86	34.39 (15.05) 9–77	22.17 (10.92) 0–42

*Notes*. Total columns are the summation of lead and assistant communications. Total Unsupportive Comments totals did not include Behavioral Control counts

^a^Breakfast mealtime data only from Arkansas^b^Not inclusive of behavioral control statements^c^Inclusive of all statement categories

Within the supportive statement category overall, the most frequently indicated statement category was Exploring Foods for Lead ECET across all mealtimes (observed 3.23, 2.70, and 1.61 times per Lunch_1_, Lunch_2_, and Breakfast, respectively), as well as for Assistants at Lunch_1_ and Breakfast (observed 2.03 and 0.89 times, respectively). However, Encourages Trying in Positive Way was the highest indicated supportive statement category at the Lunch_2_ observation (observed 1.30 times) for Assistants. The least frequently indicated supportive statement category was Hunger Cues across all mealtimes for both Lead and Assistant ECETs. Comments fitting this statement category were observed approximately 0.18 times per meal on average. Lead ECETs averaged about eight supportive comments at both Lunch mealtimes and about five at Breakfast. Assistant ECETs averaged about six (Lunch_1_), five (Lunch_2_), and three (Breakfast) supportive comments.

The most frequently indicated unsupportive statement category for both Lead and Assistant ECETs across all mealtime observations was Firm Behavioral Control. On average, comments fitting this category were observed about six times at Lunch and four times at Breakfast for Lead ECETs. Assistants averaged over six Firm Behavioral Control statements at Lunch_1_, about four at Lunch_2_ and about three at the Breakfast observation. The maximum number of Firm Behavioral Control comments made by an Assistant ECET was 26 times during a Lunch observation; 20 was the maximum for a Lead ECET during a Lunch. Across both ECET roles, comments fitting the Firm Behavioral Control statement category were observed fewer times on average for the Breakfast mealtime observation compared to the Lunch observations. The second most common unsupportive statement category observed was Pressure to Eat for all mealtimes for both Lead and Assistant ECETs. Comments fitting the Pressure to Eat statement category were observed about three times across all meals for both Lead and Assistant ECETs. Notably, the average occurrence of comments fitting the majority of all other unsupportive statement categories, besides Firm Behavioral Control, was less than once per meal. The most uncommon unsupportive statement category across both ECET types was Threats, followed by Negative Comments about the Food Served.

### Assessing Interrater reliability

Table 3 summarizes interrater reliability estimates. For commonly occurring statement categories (*N* = 11), the majority of ICCs were in the excellent range (*n* = 7, 63.7%). In addition, Positive Comments-Teacher Focus for Lead ECETs fell within the good range (ICC = .83), while Encourage Trying in a Positive Way for Lead ECETs (ICC = .64) and Exploring Foods for Assistant ECETs (ICC = .65) fell within the moderate range. Only one statement category, Encourage Trying in a Positive Way for Assistant ECETs, fell within the poor range (ICC = .14).

**Table 3:** Interrater reliability of Table Talk-Revised statement categories

ICC		Table Talk-Revised Item	Percent Agreement	
Lead *n* = 11	Assistant *n* = 6		Lead *n* = 11	Assistant *n* = 6
.83	–	Positive comments – Teacher Focus	–	67%
–	–	Positive comments – Food Focus	64%	83%
–	–	Hunger cues	91%	83%
.64	.14	Encourage trying in positive way	–	–
.91	–	Nutrition coaching	–	33%
.98	.65	Exploring foods	–	–
–	–	Negative comments about the food served	100%	83%
.95	.94	Pressure to eat	–	–
.96	–	Hurries to finish eating	–	83%
–	–	Discourage manipulating food	64%	50%
–	–	Social comparison	100%	100%
–	–	Threats	100%	100%
–	–	Preference for unhealthy food	100%	83%
–	–	Food as a reward	82%	100%
.95	.96	Focus on behavioral control	–	–

*Notes*. *ICC* Intraclass correlation coefficient. If mean of the statement category was ≥1 across the three time points for Lead/Assistant, ICC was used. If mean was < 1, percent agreement used. ICC estimates were based on a mean rating (*k* = 2), absolute agreement, one-way random-effects model

For low frequency statement categories (*N* = 19), percent agreement ranged from 33 to 100%. Almost half (*n* = 8, 42%) of the 19 values had > 90% agreement. For Lead ECETs, perfect agreement (100%) was found for Negative Comments about the Food Served, Social Comparison, Threats, and Preference for Unhealthy Food. Hunger Cues (91%) and Food as a Reward (82%) demonstrated adequate percent agreement for the Lead ECET rating pairs. For Assistant ECETs, Social Comparison, Threats, and Food as a Reward demonstrated perfect agreement (100%). Moderate agreement was found for Lead ECETs on Positive Comments-Food Focus and Discourage Manipulating Food (both 64% agreement). For Assistant ECETs, 83% agreement was found for Hurries to Finish Eating, Negative Comments about the Food Served, Hunger Cues, and Positive Comments-Food Focus, and 67% agreement was observed for Positive Comments – Teacher Focus. Only two statement categories had low agreement (< 50%), and both were for Assistant ECETs - Discourage Manipulating Food (50%) and Nutrition Coaching (33%).

### Comparing 2 lunches and breakfast

The observed counts of supportive comments were similar across the two lunch observations, with only Exploring Foods exceeding a difference of 0.25 between the two lunch occasions. Both Lead and Assistant ECETs had a higher average number of supportive comments at both Lunch observations compared to the average number at the Breakfast observation (about 6 more total positive comments at Lunch). Teacher Focus comments were more common at Breakfast than were Food Focused positive comments. For Nutrition Coaching, observers noted these comments occurring about once per Breakfast and twice per lunch observation. Negative comments across the 2 lunch observations were also similar (i.e., within 0.32 for total classroom counts) except Firm Behavioral Control. Both Lead and Assistant ECETs had a lower average number of unsupportive comments at Breakfast compared to Lunch meals.

## Discussion

This study builds on prior development work of the Table Talk tool to share descriptive data on new and existing items at multiple lunch occasions, at breakfast, and in both Head Start and state-funded preschool settings. Data suggest the additions to the TT-R capture valuable information in a reliable manner. The supportive comment additions occurred up to 14 times per meal (Food Focused Positive Comments), with the Nutrition Coaching addition occurring up to 11 times per meal. These frequencies suggest the potential salience and fit of these additions as intentional strategies used by teachers. However, inconsistency in the use of these items (min = 0; means ≈ 2) indicates an opportunity to translate these practices to other classrooms for advancing a PME [25]. In light of our prior work, [17] this may be best accomplished by integrating training and education about feeding practices and nutrition into other domains with which teachers are already familiar (e.g., social emotional development, language and literacy, school readiness). Additions to the unsupportive category also functioned well. Specifically, the additional unsupportive statement category (i.e., Social Comparisons) occurred about 0.5 times per meal (max 10 times). In general, high interrater reliability was documented for all TT-R items with the exception of 3 items for Assistant ECETs. The lower interrater reliability for these 3 items for Assistants suggests a need for increased item clarity and training materials enhancement. Overall, the study suggests utility and reliability of the revised Table Talk tool for capturing feeding communications in ECE at breakfast and lunch and for both lead and assistant teachers.

In relation to the bioecological theory, these findings speak to characteristics of the *Person, Context,* and *Time.* According to the theory, “developmentally generative” and “developmentally disruptive” personal dispositions “can set proximal processes in motion and sustain their operation. (p 795).” [26] Applying this theory to the ECE setting, ECETs who engage in high rates of pressuring children exhibit a “developmentally disruptive behavioral disposition.” [26] Conversely, frequent use of supportive practices, such as Food Focused Positive Comments and Nutrition Coaching are more representative of “developmentally generative” characteristics [26]. Professional development and policies that support the holistic growth and wellbeing of ECETs may contribute to more positive dispositions that in turn, benefit children through use of supportive practices. Within the *Context* of the classroom, children are exposed to proximal processes, which “encompass particular forms of interaction between [a child] and environment.” [26] For example, policies against ECETs sharing meals with children are a contextual factor that may influence feeding practices [17]. Over *Time*, continued exposure to these proximal processes (supportive and/or unsupportive) may function to influence development of feeding practices among children [26]. The TT-R provides a tool to measure personal ECET practices; this tool can be used to examine personal practice as a function of context and to examine the influence of ECET practices over time on the development of healthy feeding behaviors for children.

A key feature of the Table Talk tool is the ability to capture feeding communications without the limitations of floor and ceiling effects inherent in Likert-scale measurements. Illustrative of this feature and consistent with the publication of the original Table Talk tool and prior work, [27, 28] ECETs pressured children to eat frequently in this study. Our previous study documented pressure approximately every 7 min, with a maximum rate of once every 1.2 min. The current study documented Pressure to Eat approximately every 3.5 min, with a maximum of every 45 s. ECETs were greater than 16 times more likely to pressure a child to eat than to cue them to hunger and satiety. This increase is notable in the context of the broader settings included in this study versus those of the original Table Talk development. Further, this example illustrates the ability of the Table Talk tool to capture the relative frequency of feeding practices that are coercive and controlling to those that are more responsive and supportive. In fact, a high rate of unsupportive communications as captured by the Table Talk tool relative to supportive comments might indicate than an ECET is “taking excessive control of the feeding situation (p 495),” an indication of coercive feeding [26]. Keeping in mind the “interplay of *Process x Person x Context x Time*” [4], communicating supportive behaviors and practices over unsupportive ones is essential to promoting a PME over a NME. Documenting this level of specificity in differences across studies and in comparing types of feeding practices would not be possible with Likert scales.

Another feature of the Table Talk tool is the ability for use at multiple meal occasions. In this study, lunches were similar in observed communications with some notable differences from breakfast. Specifically, Lunch included more communications from ECETs than did Breakfast for both supportive and unsupportive statements despite similar length of the mealtime occasions (less than 2 min difference). Specifically, ECETs made about 45% more positive comments and 25% more negative comments at Lunch than at Breakfast. The greatest item-level discrepancies for supportive statements were observed for Exploring Foods and Encouraging Trying; greatest discrepancies for unsupportive statements were for Pressure to Eat and Firm Behavioral Control. There are several possible reasons for these observations. For example, the Breakfast meal is often a more limited menu, [29, 30] and parents are frequently dropping off during Breakfast time. These differences from Lunch may restrict ECETs’ opportunities to interact with the children. Regardless, these data suggest that Lunch and Breakfast are not interchangeable mealtime settings for the purposes of research. Further, data suggest that item counts were largely consistent across the 2 Lunch occasions with the exception of Exploring Foods and Firm Behavioral Control, which may be more sensitive to menu and daily routine differences. Taken together, data suggest that exchanging breakfast and lunch observations for intervention outcome assessment is likely to introduce bias into a study, while averaging or exchanging Lunch observations may be less problematic.

Capturing Lead and Assistant statements separately is another unique feature of the Table Talk tool. In our study, Assistant ECETs communicated less than Lead ECETs for both supportive and unsupportive statements. This finding is a difference from our prior work [16] where Assistants communicated fewer supportive statements but equal numbers of unsupportive statements. In fact, Assistant ECETs communicated 4–6 times less frequently per meal on average than did Lead ECETs for our target observed communications. This finding is expected to some degree as Lead ECETs often take responsibility for guiding children throughout the day. However, an optimal meal setting [e.g., family style in the classroom [6]] could create opportunities for each ECET to sit and talk with half the class equally. In so doing, the potential influence of each ECET could be maximized. The unequal communications also highlight an opportunity to include both Lead and Assistant ECETs in future interventions and to train them on the equal value of their role for supporting children at meals.

This study overcomes some, but not all, of the limitations identified in our prior study [16]. First, the current study expanded observations beyond HS settings. This is an important first step; expansion efforts need to be continued toward inclusion of private childcare, school-based programs, and family childcare homes. Second, the additions to the tool provided greater balance between positive and negative communications reflecting an improved understanding of input from ECETs themselves through our recent qualitative work [17, 31]. These additions extend the potential utility of Table Talk as a pragmatic tool for professionals and researchers to assist ECETs in improving their feeding practices by balancing the tool to capture both ECET strengths and areas for improvement. Third, this study included two occasions of Lunch as well as a Breakfast to show the similarity and difference between ECET communications, on average, at different mealtimes. Finally, this study, due to its focus on examining performance of the new items and similar to the original, did not have a large enough sample to explore key demographic differences in feeding communications.

## Conclusions

This study presents a revised version of the previously published Table Talk tool providing data on added and revised items, interrater reliability, and descriptive statistics by teacher and meal type while also demonstrating functionality within the theoretical framework of the bioecological model. Major revisions represented in the TT-R included refinement in capturing the type of positive comments ECETs use at meals, addition of measuring ECETs’ efforts to coach children through prior and current experiences with a food, and the inclusion of record of comparisons of children to other children or social ideals in regards to eating behaviors. These changes represent an attempt to reflect recent qualitative research in the area of ECE feeding about the intent and strategies ECETs describe deliberately deploying at meals [17]. Further, this study represents a larger sample across two states and multiple ECE settings (not just one meal at HS). Thus, this study provides an expanded tool to measure PME and NME.

Prior research has attributed variability in childcare settings to the inconsistent associations between childcare exposure and overweight and obesity [32]. Yet, a majority of interventions directed toward addressing childhood obesity focus on dietary intake of children rather than environmental factors [33]. In particular, the role of the caregiver in “shaping child eating behaviors associated with healthy body weight outcomes (p 2),” such as eating self-regulation based on recognizing internal hunger and fullness cues in infrequently a focus of intervention or policy [33]. A shift toward improving ECET feeding behaviors may help to fill the need to understand, “how childcare environments can be optimized to mitigate the risk of childhood obesity (p 10).” [32] The TT-R has potential to contribute to that line of research (and/or internal assessment) within childcare environments by identifying unsupportive practices to eliminate and supportive practices to amplify across different ECE meal occasions.

## Supplementary Information

**Additional file 1.**

## Data Availability

The datasets during and/or analysed during the current study available from the corresponding author on reasonable request.
