# Supplementary material for: Table Talk: revision of an observational tool to characterize the feeding environment in early care and education settings
Source: BMC Public Health. 2021 Jan 7;21:80. doi: 10.1186/s12889-020-10087-8 (PMC7792155; doi:10.1186/s12889-020-10087-8)
Supplement: Supplementary file 1 — Additional file 1. [file 12889_2020_10087_MOESM1_ESM.docx]

| Supplementary File 1 | | |
| --- | --- | --- |
| Comparison of the Environmental Policy and Observation Expanded Feeding Practices (EPAO-EFP) and Table Talk Revised (TTR) Properties | | |
|  | EPAO- EFP | TTR |
| Context of Development | Head Start | Head Start |
| Target for Observation | Breakfast or Lunch | Breakfast or Lunch |
| Time to Complete | Average of 23 min | Average of 24 min + 15 min for classroom assimilation |
| Format | Observation Checklist | Observation Counts |
| Measurement Scale | Either Yes/No or No/1-2 times/3+ times | Continuous |
| Length | 47 items | 15 items |
| Practices Documented | Healthful Practices *(example items)*   - Talked with the children about foods they were eating - Encouraged children to try foods on their plate - Praised a child for trying new or less preferred foods - Enthusiastically role modeled eating healthy foods - Asked child if he/she was full before removing the plate - Served seconds only after a child requested seconds and the teacher asked the child if he/she was still hungry     Controlling Practices *(example items)*   - Used food as reward - Used food to control child's emotions - Pressured child to eat more than they seemed to want - Promised something other than food for eating specific food - Used food as reward for eating a specific food - Allows child to continue to take multiple servings, even if not consuming | Supportive Communications *(all items)*   - Positive comments, teacher opinion - Positive comments, teacher opinion, food facts - Use of hunger cues - Encourage trying in supportive way - Nutrition Coaching - Encouraging food exploration   Detrimental Communications *(all items)*   - Negative comments about food - Pressure to eat - Hurries to eat - Discourage food manipulation - Social comparisons - Threats - Indicates preferences for unhealthy foods - Offers food as reward - Controlling behavior |
| Strengths | - Studies published on Head Start in Rhode Island for comparison (put cites here when number settle in paper) - Some overlap between EPAO self-report and EPAO observational items to facilitate comparisons - Additional companion modules | - Captures all behaviors (no ceiling) - Sensitive to change, useful for intervention assessment - Describe magnitude and prevalence of observed behaviors - Captures behaviors of both teachers in a classroom - High level of specificity |
| Limitations | - Preset coding categories limits ability to capture variability (i.e., frequency beyond 3+) | - Not suited for traditional psychometric analysis (e.g., internal consistency) due to nature of continuous capture of individual communications |
| Inter-rater Agreement Range | - Strong reported levels of overall initial internal consistency based on 9 cases (Kappa = 0.83) and later internal consistency based on two cases (Kappa = 0.84)] | - For commonly occurring statement categories (*N* = 11), the majority of ICCs were in the excellent range. For low frequency statement categories (*N* = 19), percent agreement ranged from 33% to 100%. Almost half of the 19 values had > 90% agreement. |
|  |  |  |
| Training | - Staff Mealtime Behaviors Training Manual, adapted from the EPAO provides guide to code feeding practices | - 2 hour workshop - Video training to reliability - Confirmation of live reliability |
